# Identification of Key Pathways and Genes in Advanced Coronary Atherosclerosis Using Bioinformatics Analysis

**DOI:** 10.1155/2017/4323496

**Published:** 2017-10-31

**Authors:** Xiaowen Tan, Xiting Zhang, Lanlan Pan, Xiaoxuan Tian, Pengzhi Dong

**Affiliations:** ^1^Tianjin State Key Laboratory of Modern Chinese Medicine, Tianjin University of Traditional Chinese Medicine, Tianjin, China; ^2^Research and Development Center of TCM, Tianjin International Joint Academy of Biotechnology & Medicine, Tianjin, China

## Abstract

**Background:**

Coronary artery atherosclerosis is a chronic inflammatory disease. This study aimed to identify the key changes of gene expression between early and advanced carotid atherosclerotic plaque in human.

**Methods:**

Gene expression dataset GSE28829 was downloaded from Gene Expression Omnibus (GEO), including 16 advanced and 13 early stage atherosclerotic plaque samples from human carotid. Differentially expressed genes (DEGs) were analyzed.

**Results:**

42,450 genes were obtained from the dataset. Top 100 up- and downregulated DEGs were listed. Functional enrichment analysis and Kyoto Encyclopedia of Genes and Genomes (KEGG) identification were performed. The result of functional and pathway enrichment analysis indicted that the immune system process played a critical role in the progression of carotid atherosclerotic plaque. Protein-protein interaction (PPI) networks were performed either. Top 10 hub genes were identified from PPI network and top 6 modules were inferred. These genes were mainly involved in chemokine signaling pathway, cell cycle, B cell receptor signaling pathway, focal adhesion, and regulation of actin cytoskeleton.

**Conclusion:**

The present study indicated that analysis of DEGs would make a deeper understanding of the molecular mechanisms of atherosclerosis development and they might be used as molecular targets and diagnostic biomarkers for the treatment of atherosclerosis.

## 1. Introduction

Atherosclerosis associated cardiovascular diseases (CVD) are the leading cause of mortality worldwide. Immune system responses play a pivotal role in all phases of atherosclerosis [1] and inflammation responses contribute to focal plaque vulnerability [2]. High-level LDL in plasma and other atherosclerosis-prone conditions expedite immune cell recruitment into the lesion area in the early and advanced stages [3–5]. Variety of inflammatory process was identified during atherosclerosis progression, which might be amenable to interventions.

High-throughput platforms for analysis of gene expression, such as microarrays, are the promising tools for inferring biological relevancy, especially complex network during the process of atherosclerosis. Recently, atherosclerotic gene expression profiling studies have been performed by microarray technology and suggested that hundreds of differentially expressed genes (DEGs) are involved in variety pathways, biological processes, or molecular functions. Microarray technology combined bioinformatics analysis made it possible to analyze the expression changes of mRNA from early to advanced stage of coronary atherosclerosis development, comprehensively. Samples from early ((pathological) intimal thickening and intimal xanthoma) and from advanced (thin or thick fibrous cap atheroma) lesions have been retrieved from the Maastricht Pathology Tissue Collection (MPTC) [6]. However, the protein-protein interactions (PPI) network among DEGs remains to be elucidated.

In this study, the original data was downloaded from Gene Expression Omnibus (GEO). DEGs from early and advanced lesions were screened. Subsequently, the gene ontology and biological function annotation were performed followed by PPI network analysis. By using the bioinformatic method, further investigation on mechanism of atherosclerosis was lighted and it might provide potential biomarker candidates for clinical use and drug targets discovery.

## 2. Materials and Methods

### 2.1. Microarray Data

The gene expression profiles of GSE28829 were downloaded from Gene Expression Omnibus (GEO). GSE28829 was performed on GPL570, [HG-U133_Plus_2] Affymetrix Human Genome U133 Plus 2.0 Array. The GSE28829 data set contained 29 samples, including 16 advanced atherosclerotic plaque samples and 13 early atherosclerotic plaque samples.

### 2.2. Identification of Differentially Expression Genes (DEGs)

The analysis was carried out by Morpheus (https://software.broadinstitute.org/morpheus/).  The expression files were uploaded. Advanced and early stages of atherosclerotic plaque were assigned according to the annotation of the GSE28829 (https://www.ncbi.nlm.nih.gov/geo/query/acc.cgi?acc=GSE28829). DEGs were identified using signal to noise method where a total of 42,450 genes were analyzed and top 100 (top 100 upregulated and top 100 downregulated genes) genes were listed.

### 2.3. Gene Ontology and Pathway Enrichment Analysis of DEGs

Cellular component, molecular function, biological process, and Kyoto Encyclopedia of Genes and Genomes (KEGG) were analyzed using a web-based tool, search tool for the retrieval of interacting genes (STRING) (https://string-db.org/). Due to limitation of the settings of the tool, top 2000 upregulated genes and top 2000 downregulated genes were analyzed.

### 2.4. Integration of Protein-Protein Interaction (PPI) Network and Module Analysis

STRING (version 10.0) was used to evaluate the interactive (PPI) relationships between DEGs. Only experimentally validated interactions with a combined score >0.4 were selected as significant. PPI networks were constructed using the Cytoscape software. A plug-in molecular complex detection (MCODE) was used to screen the modules of PPI network identified in Cytoscape. Modules inferred using the default settings that the degree cutoff was set at 2, node score cutoff was set at 0.2, *K*-core was set at 2, and max. depth was 100.

### 2.5. Pathways Interrelation Analysis

Pathways interrelation analysis was carried out using plug-in ClueGO v2.3.3. Genes composed of modules A and D (inferred from MCODE) were analyzed. KEGG was conducted and pathways with *P* < 0.05 were showed in Figure 3.

## 3. Results

### 3.1. Identification of Differentially Expressed Genes (DEGs)

**Figure 1:**
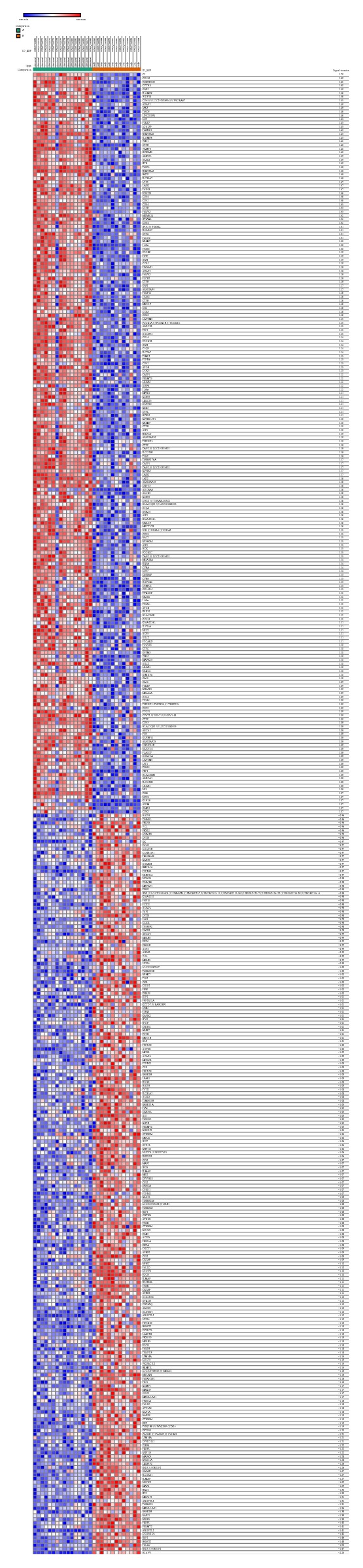
Heat map of the top 100 differentially expressed genes between advanced and early atherosclerosis (100 upregulated genes and 100 downregulated genes). Red: upregulation; purple: downregulation.

29 samples from atherosclerotic carotid artery segments, 16 advanced and 13 early lesions included, have been retrieved from the Maastricht Pathology Tissue Collection (MPTC). The series from each chip was analyzed by Morpheus using signal to noise method to find out as much as possible genes up- or downregulated. Among the total 42,450 genes, the most significant signal of upregulated gene is C2, and the signal to noise score is 1.792. The most significant signal of downregulated gene is H2AFV where the signal to noise score is −2.249. All the DEGs were listed (data not shown). Top 100 upregulated and downregulated genes were listed, as shown in Figure 1.

### 3.2. Gene Ontology and Pathway Enrichment Analysis

Due to the limited number of nods of the tool, we selected the top 2,000 DEGs, 2000 up- and downregulated genes, respectively. Top 5 enrichment analyses were showed for each part of gene ontology (GO) analysis. The results showed that the upregulated genes significantly took part in the formation of cellular components (GO) that were lysosome (GO.0005764), vacuole (GO.0005773), plasma membrane (GO.0005886), cell periphery (GO.0071944), and plasma membrane part (GO.0044459). Downregulated genes were mainly involved in construction of cytoplasm (GO.0005737), intracellular organelle (GO.0043229), organelle part (GO.0044422), cytoplasmic part (GO.0044444), and intracellular organelle part (GO.0044446), as shown in Table 1. The molecular function (GO) enrichment analysis showed that the upregulated genes were mainly involved in protein binding (GO.0005515), receptor binding (GO.0005102), molecular transducer activity (GO.0060089), molecular function (GO.0003674), and binding (GO.0005488). Downregulated genes mainly revolved in protein binding (GO.0005515), binding (GO.0005488), cytoskeletal protein binding (GO.0008092), enzyme binding (GO.0019899), and nucleotide binding (GO.0000166) as shown in Table 2. Biological process enrichment analysis showed that upregulated genes take part in the immune system process (GO.0002376), defense response (GO.0006952), regulation of immune system process (GO.0002682), immune response (GO.0006955), and regulation of immune response (GO.0050776). Downregulated genes take part in cytoskeleton organization (GO.0007010), cellular component organization (GO.0016043), positive regulation of cellular process (GO.0048522), regulation of cellular component organization (GO.0051128), and cellular component organization or biogenesis (GO.0071840) as shown in Table 3. Kyoto Encyclopedia of Genes and Genomes (KEGG) pathways enrichment analysis was conducted where the upregulated genes are enriched in osteoclast differentiation (4380), cytokine-cytokine receptor interaction (4060), chemokine signaling pathway (4062), lysosome (4142), and* Staphylococcus aureus* infection (5150). Downregulated genes are enriched in focal adhesion (4510), regulation of actin cytoskeleton (4810), arrhythmogenic right ventricular cardiomyopathy (ARVC) (5412), oxytocin signaling pathway (4921), and cGMP-PKG signaling pathway (4022).

### 3.3. Module Screening from the Protein-Protein Interaction (PPI) Network

**Figure 2:**
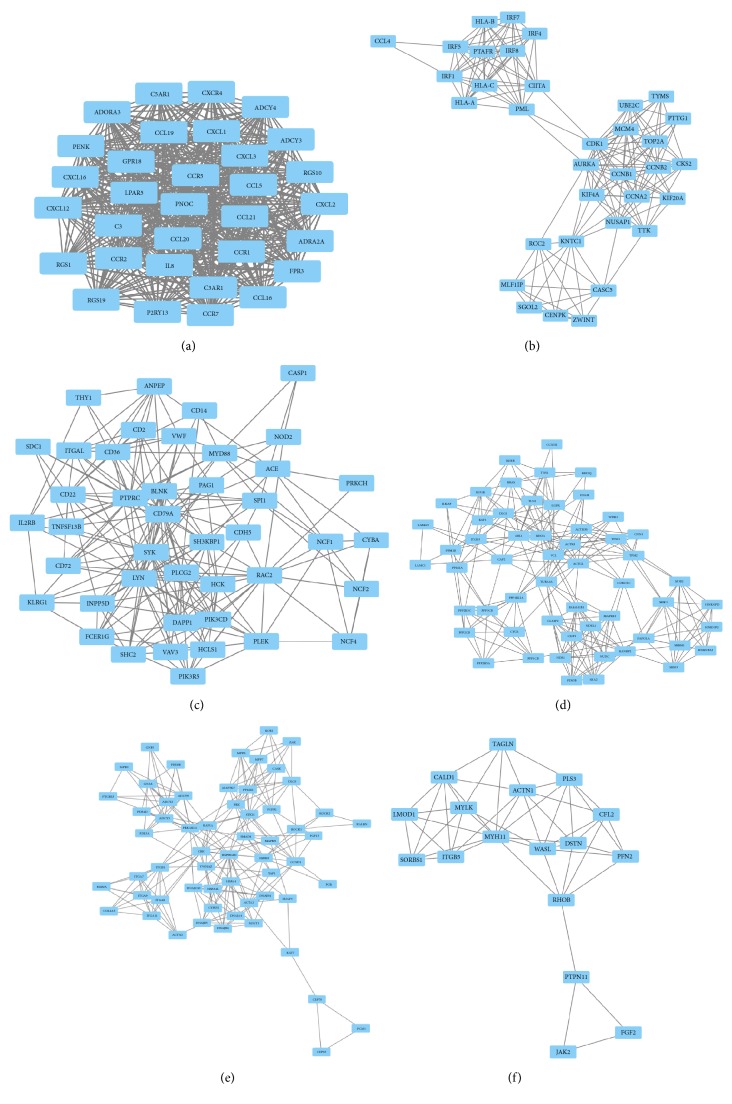
Top 6 modules from the protein-protein interaction network. (a) module 1; (b) module 2; (c) module 3; (d) module 4; (e) module 5; (f) module 6.

Based on the information in the STRING database, top 10 hub genes were screened. These genes are ubiquitin A-52 residue ribosomal protein fusion product 1 (UBA52), ribosomal protein L38 (RPL38), integrin subunit alpha L (ITGAL), intercellular adhesion molecule 1 (ICAM1), interleukin 7 receptor (IL7R), interleukin 7 (IL7), REL protooncogene, NF-KB subunit (REL), NF-KB inhibitor alpha (NFKBIA), Vav guanine nucleotide exchange factor 1 (VAV1), and lymphocyte cytosolic protein 2 (LCP2). 2693 nods and 9212 edges were analyzed using the plug-in MCODE in Cytoscape. The top 6 significant modules were selected; modules A, B, and C were inferred from upregulated genes while modules D, E, and F were inferred from downregulated genes, and the functional annotation of the genes involved in the modules was analyzed (as shown in Figure 2). Enrichment analysis showed that the genes in module were mainly associated with chemokine signaling pathway, cell cycle, B cell receptor signaling pathway focal adhesion, and regulation of actin cytoskeleton. Those genes involved in inferred modules were listed in Table 5.

### 3.4. Pathways Interrelation Analysis

**Figure 3:**
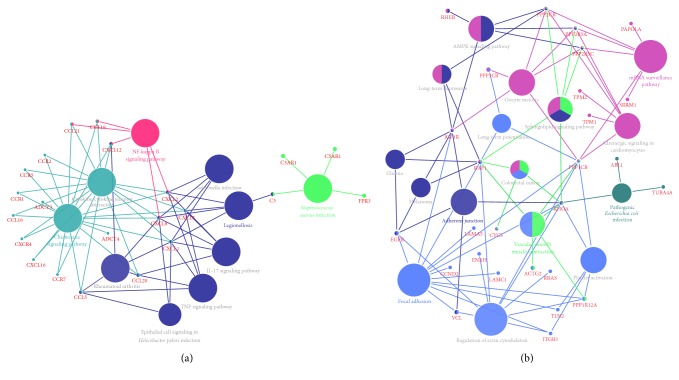
Interrelation between pathways. (a) Interrelation inferred from module A. (b) Interrelation inferred from module D.

In order to investigate the involved interrelation between the pathways unidentified before, modules inferred from the network were analyzed and the interrelation between pathways and genes involved was drawn as shown in Figure 3. Modules with highest MCODE score were selected where for module A inferred from upregulated DEGs and module D from downregulated DEGs (Figure 2) pathways interrelation analysis was conducted. As shown in Figure 3(a), these genes from module A mainly are involved in four pathways that were NF-kappa B signaling pathway, chemokine signaling pathway, legionellosis signaling pathway (with* Salmonella* infection, interleukin 17 (IL-17), tumor necrosis factor (TNF), epithelial cell, and rheumatoid arthritis (RA) signaling pathway as subgroups), and* Staphylococcus aureus* infection signaling pathway. C-X-C motif chemokine ligand 2 (CXCL2), C-X-C motif chemokine ligand 3 (CXCL3), C-X-C motif chemokine ligand 8 (CXCL8), and C-X-C motif chemokine ligand 12 (CXCL12) took part in three pathways which were NF-kappa B signaling pathway, chemokine signaling pathway, and legionellosis signaling pathway (nodes in three colors). C-C motif chemokine ligands 19 and 21 (CCL19, CCL21) were involved in NF-kappa B and chemokine signaling pathway while C-C motif chemokine ligand 5 (CCL5), C-C motif chemokine ligand 20 (CCL20), C-X-C motif chemokine ligand 1 (CXCL1), and C-X-C motif chemokine ligand 1 (CXCL3) played a role in both legionellosis and chemokine signaling pathway. Besides, Complement C3 (C3) participated in legionellosis and* Staphylococcus aureus* infection signaling pathway. Pathway and gene set were listed in Table 6. Analysis of module D demonstrated that these genes were mainly involved in focal adhesion (with regulation of actin cytoskeleton, platelet activation, and long-term potentiation as subgroups), adherens junction (with glioma, melanoma signaling pathways as subgroups), pathogenic* Escherichia coli* infection, and mRNA surveillance pathway (with adrenergic signaling in cardiomyocytes, oocytes meiosis signaling pathway as subgroups). Among these genes, RHOA took in 5 pathways that were pathogenic* Escherichia coli* infection, vascular smooth muscle contraction, focal adhesion, adherens junction, and mRNA surveillance pathway. Raf-1 protooncogene and serine/threonine kinase (RAF1) participate in 4 pathways. Protein phosphatase 2 catalytic subunit beta (PPP2CB), protein phosphatase 2 regulatory subunit B (B56) alpha isoform (PPP2R5A), protein phosphatase 2 regulatory subunit B (B56) gamma isoform (PPP2R5C), protein phosphatase 1 catalytic subunit beta (PPP1CB), insulin-like growth factor 1 receptor (IGF1R) and cytochrome C, somatic (CYCS), participate in 3 pathways. Ras homolog enriched in brain (RHEB), protein phosphatase 3 catalytic subunit beta (PPP3CB), epidermal growth factor receptor (EGFR), smooth muscle gamma-actin (ACTG2), Vinculin (VCL), and protein phosphatase 1 regulatory subunit 12A (PPP1R12A) took part in 2 pathways. Pathway and gene set were listed in Table 7.

## 4. Discussion

The underlying cause of the cardiovascular event is atherosclerosis, a chronic inflammatory disease [7]. Profoundly understanding the molecular mechanism of atherosclerosis was critically important for diagnosis and treatment of cardiovascular disease. Since microarray and high-throughput sequencing provided thousands of gene expression data types, it has been widely used to predict the potential therapeutic targets for atherosclerosis. In the present study, GSE28829 was analyzed and the total differentially expressed genes were identified between early and advanced plaque collected from patients. Functional annotation demonstrated that these DEGs were mainly involved in osteoclast differentiation, cytokine-cytokine receptor interaction, chemokine signaling pathway, lysosome and* Staphylococcus aureus* infection, focal adhesion, regulation of actin cytoskeleton, arrhythmogenic right ventricular cardiomyopathy (ARVC), oxytocin signaling pathway, and cGMP-PKG signaling pathway.

Cross-talks between the vascular and immune system play a critical role in atherosclerosis. It is a key point that new drug development should not be focused on cardiovascular system only; the immune system is the potential target for the treatment of atherosclerosis either. The osteoclast-associated receptor (OSCAR), originally described in bone as immunological mediator and regulator of osteoclast differentiation, may be involved in cell activation and inflammation during atherosclerosis [8]. Cytokine interactions mainly involved interleukins (IL), transforming growth factors (TGF), interferons (IFN), and tumor necrosis factors (TNF) [9, 10]. CCL2, CCL5, IFN-*γ*, and TNF-*α* participate in the monocyte recruitment. IFN-*γ*, IL-1*β*, TGF-*β*, and TNF-*α* take part in plaque stability. IFN-*γ*, IL-1*β*, IL-6, IL-12, IL-33, and M-CSF are involved in lesion formation. These signaling pathways but also those identified in this study are well documented where these cytokine targeted therapies use antibodies to block and inhibit proinflammatory cytokine signaling in order to dampen the inflammatory response observed in atherosclerotic lesions [11]. In this study, signal to noise method implanted in the Morpheus was used to identify the DEGs where this method could get most number of DEGs. In order to better understand the interaction of DEGs, GO and KEGG analysis were performed.

The GO term analysis revealed that the upregulated genes were mainly involved in immune system process, defense response, and regulation of immune system process (Table 3). These results showed that, as atherosclerosis developed, immune system cells activated and gathered in the plaque [12–14]. Downregulated genes were mainly involved in cytoskeleton organization, cellular component organization, and positive regulation of cellular process and confirm the recent findings [15–17] (Table 3). Besides, as shown in Table 4, the KEGG analysis showed that upregulated genes participate in osteoclast differentiation [18–20], cytokine-cytokine receptor interaction [21], and chemokine signaling pathway [22–24]. Downregulated genes took part in focal adhesion [25–27], regulation of actin cytoskeleton [27–29], and arrhythmogenic right ventricular cardiomyopathy (ARVC) [30]. These pathways demonstrated promising targets for new drugs intervention. It is important to keep in mind that the upstream or the key node gene might not be the appropriate target for drug design because of the core effects and far-range effects especially the side effects that prevent the further application of the drugs. These GO term and KEGG analyses indicated the possible direction of experimental validation.

Next, the protein-protein interaction (PPI) network was evaluated and top degree hub genes were listed: ubiquitin A-52 residue ribosomal protein fusion product 1 (UBA52), ribosomal protein L38 (RPL38), integrin subunit alpha L (ITGAL), intercellular adhesion molecule 1 (ICAM1), interleukin 7 receptor (IL7R), interleukin 7 (IL7), REL protooncogene, NF-KB subunit (REL) and NF-KB inhibitor alpha (NFKBIA), Vav guanine nucleotide exchange factor 1 (VAV1), and lymphocyte cytosolic protein 2 (LCP2). The most significant hub gene in the network is UBA52. UBA52 regulates ubiquitination of ribosome and sustains embryonic development [31]. RPL38 takes part in RNA binding [32] and constructing ribosome [33]. ITGAL contributes to natural killer cell cytotoxicity [34], involved in leukocyte adhesion and transmigration of leukocytes [35]. ICAM1 acts as a receptor for major receptor group rhinovirus A-B capsid proteins [36, 37]. As Kaposi's sarcoma-associated herpesvirus/HHV-8 infection, ICAM1 is degraded by viral E3 ubiquitin ligase MIR2, presumably to prevent lysis of infected cells by cytotoxic T-lymphocytes and NK cell [38]. IL7R, a secreted protein, is not only the receptor of interleukin 7 (IL7) but also the receptor for thymic stromal lymphopoietin (TSLP). IL7 stimulates the proliferation of lymphoid progenitor cells and B cell maturation [39–42]. REL plays a role in differentiation and lymphopoiesis that formed heterodimer (or homodimer) to help translocation of NF-kappa B [43, 44]. Interestingly, the inhibitor of NF-kappa B complex translocation, NFKBIA, was induced either, where this gene traps the REL dimers in cytoplasm by masking the nuclear translocation signals [45, 46]. VAV1 is another critical transducer of T cell receptor signals to the calcium and extracellular signal-regulated kinases (ERK) pathways [47, 48]. Lastly, LCP2 is involved in T cell antigen receptor mediated signaling [47]. In conclusion, these hub genes are mainly involved in immune systems cells recruitment in the plaque, such as T cells and B cells gathering.

PPI network analysis demonstrated that both up- and downregulated genes interacted directly or indirectly. The more edges associated with genes indicated the more potential selection for the targets. Given the fact that PPI is considered a new type of targets, appropriate methods for screening are pivotal for drug development. Förster resonance energy transfer (FRET) and fluorescence lifetime microscopy (FLIM) are useful cell-based methods for high-throughput screening (HTS). Based on our findings, expression vectors of interactive protein can be constructed for drug screening. For example, REL and NFKBIA can be cotransfected into cells and screen the molecules that inhibit or activate the interaction between the proteins.

Module analysis of the PPI network showed that the development of atherosclerosis was associated with chemokine signaling pathway, cell cycle, B cell receptor signaling pathway, focal adhesion, and regulation of actin cytoskeleton. Indeed, kinds of chemokines were secreted and trapped different types of immune cells to the arterial plaque [49, 50]. As atherosclerosis developed, the immune system offers a large variety of immune checkpoint proteins; both costimulatory and inhibitory proteins are involved. Costimulatory proteins can promote cell survival, cell cycle progression, and differentiation to effector and memory cells, whereas inhibitory proteins terminate these processes to halt ongoing inflammation [51]. Studies showed that B1 cells can prevent lesion formation, whereas B2 cells have been suggested to promote it [52, 53]. These activated signaling pathways are key to the development of atherosclerosis; it suggested the promising candidates for therapeutic intervention.

Interrelation between pathway showed that cross-talk arises through genes participating in different signaling pathways. It was suggested that these genes might be used as targets for intervention.

Liver X receptors (LXRs), as a promising target, preventing the development of atherosclerosis, attracted much more attention during these years. Both activators of LXR*α* and LXR*β* presented preferable effects in preclinical practice but due to unclarified mechanism, these activators always induce adverse neurological events [54, 55]. Analysis of interrelation between pathways suggested that the fact that the cross-talk might be beneficial or detrimental for the ultimate clinical goal should be taken much more into consideration.

## 5. Conclusion

All these results in this study inspired that immune system and inflammation progress are the promising targets for prevention of atherosclerosis besides lipid lowering and cholesterol metabolism regulation. In fact, immune system disorders are the physiological and pathological basis of many diseases, including angiocardiopathy [56–59]. Our data provides a comprehensive bioinformatics analysis of DEGs that might be involved in the development of atherosclerosis. Those genes and signaling pathway identified in this study implied further application for clinical use. However, molecular biological experiments are required to confirm the function of the identified genes in atherosclerosis.

## Figures and Tables

**Table 1:** Cellular component (GO) enrichment analysis in networks.

Expression	#Pathway ID	Pathway description	Observed gene count	False discovery rate
Upregulated	GO.0005764	Lysosome	103	1.02*E* − 25
	GO.0005773	Vacuole	112	1.32*E* − 25
	GO.0005886	Plasma membrane	441	1.46*E* − 25
	GO.0071944	Cell periphery	446	1.46*E* − 25
	GO.0044459	Plasma membrane part	268	1.30*E* − 24

Downregulated	GO.0005737	Cytoplasm	807	9.90*E* − 37
	GO.0043229	Intracellular organelle	838	1.42*E* − 26
	GO.0044422	Organelle part	639	3.03*E* − 26
	GO.0044444	Cytoplasmic part	611	3.03*E* − 26
	GO.0044446	Intracellular organelle part	628	3.03*E* − 26

**Table 2:** Molecular function (GO) enrichment analysis in networks.

Expression	#Pathway ID	Pathway description	Observed gene count	False discovery rate
Upregulated	GO.0005515	Protein binding	445	7.57*E* − 24
	GO.0005102	Receptor binding	148	1.76*E* − 16
	GO.0060089	Molecular transducer activity	194	1.39*E* − 14
	GO.0003674	Molecular_function	889	4.21*E* − 12
	GO.0005488	Binding	761	4.21*E* − 12

Downregulated	GO.0005515	Protein binding	426	8.46*E* − 22
	GO.0005488	Binding	740	3.00*E* − 12
	GO.0008092	Cytoskeletal protein binding	79	7.43*E* − 12
	GO.0019899	Enzyme binding	151	7.43*E* − 12
	GO.0000166	Nucleotide binding	218	1.07*E* − 08

**Table 3:** Biological process (GO) enrichment analysis in networks.

Expression	#Pathway ID	Pathway description	Observed gene count	False discovery rate
Upregulated	GO.0002376	Immune system process	321	7.44*E* − 63
	GO.0006952	Defense response	252	5.46*E* − 56
	GO.0002682	Regulation of immune system process	246	8.58*E* − 56
	GO.0006955	Immune response	241	2.11*E* − 55
	GO.0050776	Regulation of immune response	175	4.95*E* − 49

Downregulated	GO.0007010	Cytoskeleton organization	109	2.81*E* − 12
	GO.0016043	Cellular component organization	386	2.81*E* − 12
	GO.0048522	Positive regulation of cellular process	362	2.81*E* − 12
	GO.0051128	Regulation of cellular component organization	211	6.71*E* − 12
	GO.0071840	Cellular component organization or biogenesis	390	1.02*E* − 11

**Table 4:** KEGG pathways enrichment analysis in networks.

Expression	#Pathway ID	Pathway description	Observed gene count	False discovery rate
Upregulated	4380	Osteoclast differentiation	47	6.30*E* − 22
	4060	Cytokine-cytokine receptor interaction	63	6.90*E* − 18
	4062	Chemokine signaling pathway	51	6.91*E* − 18
	4142	Lysosome	41	1.75*E* − 17
	5150	*Staphylococcus aureus* infection	27	2.52*E* − 17

Downregulated	4510	Focal adhesion	38	3.66*E* − 07
	4810	Regulation of actin cytoskeleton	37	1.46*E* − 06
	5412	Arrhythmogenic right ventricular cardiomyopathy (ARVC)	20	1.52*E* − 06
	4921	Oxytocin signaling pathway	30	1.58*E* − 06
	4022	cGMP-PKG signaling pathway	30	1.95*E* − 06

**Table 5:** Top 6 modules from protein-protein interaction network.

Gene set	MCODE score	Nodes
Chemokine signaling pathway	31.419	CCL20, PNOC, PENK, LPAR5, C5AR1, CCR1, RGS1, ADRA2A, CXCL3, CXCL2, CCL21, CCR7, CCL19, FPR3, CCR5, CCL5, CXCL16, ADCY3, P2RY13, ADORA3, CCL16, IL8, RGS10, RGS19, CXCL1, C3, CCR2, C3AR1, CXCL12, GPR18, CXCR4, ADCY4

Cell cycle	10.303	KIF20A, PTTG1, HLA-B, CDK1, CASC5, IRF8, IRF4, CIITA, CKS2, IRF5, IRF1, IRF7, CCL4, UBE2C, KIF4A, CCNA2, AURKA, ZWINT, TOP2A, HLA-A, PTAFR, TYMS, CENPK, CCNB1, MLF1IP, HLA-C, NUSAP1, KNTC1, SGOL2, MCM4, PML, RCC2, CCNB2, TTK

B cell receptor signaling pathway	9.762	VWF, NCF4, SH3KBP1, PLEK, CD14, TNFSF13B, CD79A, BLNK, CD72, SPI1, PTPRC, KLRG1, IL2RB, PIK3CD, SYK, VAV3, CASP1, CDH5, PLCG2, FCER1G, SDC1, NCF1, INPP5D, PIK3R5, RAC2, CD22, NCF2, LYN, ACE, CYBA, DAPP1, ITGAL, HCLS1, MYD88, PRKCH, PAG1, ANPEP, CD36, THY1, SHC2, NOD2, HCK, CD2

Focal adhesion	8.852	TNS1, DLG1, NDE1, PPM1B, WDR1, RRAS, ITGB3, CNN1, SRSF1, CCND2, HNRNPD, IGF1R, TPM1, TPM2, EGFR, RHEB, TLN2, HNRNPU, PDS5B, SKA2, NUDC, PAFAH1B1, RHOQ, ABL1, CAP2, ACTG2, PPP3CB, TUBA4A, CORO1C, PAPOLA, RAF1, LAMA5, SF3B2, LAMC1, MAPRE1, ILKAP, RHOA, NDEL1, SRRM1, SRSF3, PPM1A, PPP2R5A, PPP1R12A, PPP1CB, ACTR3B, HNRNPA3, CYCS, ENAH, CLIP1, CLASP2, PPP2CB, RANBP2, PPP2R5C, VCL, ACTR3

Focal adhesion	7.414	YWHAZ, ITGA11, GNB5, KALRN, ROCK1, DNAJB4, SMAD4, S1PR3, DNAJC10, ITGB1, DNAJB6, ITGA8, ERBB2, FGF13, PDE4D, GNAS, PRKAR1A, CYFIP2, FRK, DNAJB5, CCND1, CASK, CRK, ADCY9, DLG3, HSPA4, HSPA4L, ADCY5, H2AFV, PDE3A, YAP1, PGR, MPP7, ITGA9, ROCK2, PDE8B, PCM1, PARVA, ADCY2, RAP1A, KAT7, ITGA7, DNAJA4, MAPK9, ACTN2, ZAK, CEP70, CEP63, PPM1K, MAP3K7, MPP6, COL4A5, HSP90AB1, SUGT1, STK11, PTGER3, FGFR1, ACTA2, ROR1

Regulation of actin cytoskeleton	6.25	CALD1, PLS3, FGF2, TAGLN, RHOB, CFL2, MYLK, ITGB5, WASL, JAK2, LMOD1, DSTN, PFN2, MYH11, ACTN1, PTPN11, SORBS1

**Table 6:** KEGG analysis based on module A.

GOID	GO term	Ontology source	Term *P* value	Nr. genes	Associated genes found
GO:0004064	NF-kappa B signaling pathway	KEGG_01.03.2017	19.0*E* − 6	5.00	[CCL19, CCL21, CXCL12, CXCL2, CXCL8]

GO:0005150	*Staphylococcus aureus* infection	KEGG_01.03.2017	43.0*E* − 6	4.00	[C3, C3AR1, C5AR1, FPR3]

GO:0004060	Cytokine-cytokine receptor interaction	KEGG_01.03.2017	37.0*E* − 18	16.00	[CCL16, CCL19, CCL20, CCL21, CCL5, CCR1, CCR2, CCR5, CCR7, CXCL1, CXCL12, CXCL16, CXCL2, CXCL3, CXCL8, CXCR4]

GO:0004062	Chemokine signaling pathway	KEGG_01.03.2017	10.0*E* − 24	18.00	[ADCY3, ADCY4, CCL16, CCL19, CCL20, CCL21, CCL5, CCR1, CCR2, CCR5, CCR7, CXCL1, CXCL12, CXCL16, CXCL2, CXCL3, CXCL8, CXCR4]

GO:0004657	IL-17 signaling pathway	KEGG_01.03.2017	17.0*E* − 6	5.00	[CCL20, CXCL1, CXCL2, CXCL3, CXCL8]

GO:0004668	TNF signaling pathway	KEGG_01.03.2017	35.0*E* − 6	5.00	[CCL20, CCL5, CXCL1, CXCL2, CXCL3]

GO:0005120	Epithelial cell signaling in *Helicobacter pylori* infection	KEGG_01.03.2017	1.7*E* − 3	3.00	[CCL5, CXCL1, CXCL8]

GO:0005132	*Salmonella* infection	KEGG_01.03.2017	230.0*E* − 6	4.00	[CXCL1, CXCL2, CXCL3, CXCL8]

GO:0005134	Legionellosis	KEGG_01.03.2017	1.2*E* − 6	5.00	[C3, CXCL1, CXCL2, CXCL3, CXCL8]

GO:0005323	Rheumatoid arthritis	KEGG_01.03.2017	14.0*E* − 6	5.00	[CCL20, CCL5, CXCL1, CXCL12, CXCL8]

**Table 7:** KEGG analysis based on module D.

GOID	GO term	Ontology source	Term *P* value	Nr. genes	Associated genes found
GO:0005130	Pathogenic *Escherichia coli* infection	KEGG_01.03.2017	3.6*E* − 3	3.00	[ABL1, RHOA, TUBA4A]

GO:0004071	Sphingolipid signaling pathway	KEGG_01.03.2017	500.0*E* − 6	5.00	[PPP2CB, PPP2R5A, PPP2R5C, RAF1, RHOA]

GO:0004270	Vascular smooth muscle contraction	KEGG_01.03.2017	570.0*E* − 6	5.00	[ACTG2, PPP1CB, PPP1R12A, RAF1, RHOA]

GO:0005210	Colorectal cancer	KEGG_01.03.2017	4.6*E* − 3	3.00	[CYCS, RAF1, RHOA]

GO:0004270	Vascular smooth muscle contraction	KEGG_01.03.2017	570.0*E* − 6	5.00	[ACTG2, PPP1CB, PPP1R12A, RAF1, RHOA]

GO:0004510	Focal adhesion	KEGG_01.03.2017	560.0*E* − 12	12.00	[CCND2, EGFR, IGF1R, ITGB3, LAMA5, LAMC1, PPP1CB, PPP1R12A, RAF1, RHOA, TLN2, VCL]

GO:0004611	Platelet activation	KEGG_01.03.2017	610.0*E* − 6	5.00	[ITGB3, PPP1CB, PPP1R12A, RHOA, TLN2]

GO:0004720	Long-term potentiation	KEGG_01.03.2017	6.3*E* − 3	3.00	[PPP1CB, PPP3CB, RAF1]

GO:0004810	Regulation of actin cytoskeleton	KEGG_01.03.2017	2.1*E* − 6	9.00	[EGFR, ENAH, ITGB3, PPP1CB, PPP1R12A, RAF1, RHOA, RRAS, VCL]

GO:0005210	Colorectal cancer	KEGG_01.03.2017	4.6*E* − 3	3.00	[CYCS, RAF1, RHOA]

GO:0004071	Sphingolipid signaling pathway	KEGG_01.03.2017	500.0*E* − 6	5.00	[PPP2CB, PPP2R5A, PPP2R5C, RAF1, RHOA]

GO:0004152	AMPK signaling pathway	KEGG_01.03.2017	570.0*E* − 6	5.00	[IGF1R, PPP2CB, PPP2R5A, PPP2R5C, RHEB]

GO:0004520	Adherens junction	KEGG_01.03.2017	700.0*E* − 6	4.00	[EGFR, IGF1R, RHOA, VCL]

GO:0004730	Long-term depression	KEGG_01.03.2017	4.6*E* − 3	3.00	[IGF1R, PPP2CB, RAF1]

GO:0005214	Glioma	KEGG_01.03.2017	5.6*E* − 3	3.00	[EGFR, IGF1R, RAF1]

GO:0005218	Melanoma	KEGG_01.03.2017	6.9*E* − 3	3.00	[EGFR, IGF1R, RAF1]

GO:0003015	mRNA surveillance pathway	KEGG_01.03.2017	10.0*E* − 6	6.00	[PAPOLA, PPP1CB, PPP2CB, PPP2R5A, PPP2R5C, SRRM1]

GO:0004071	Sphingolipid signaling pathway	KEGG_01.03.2017	500.0*E* − 6	5.00	[PPP2CB, PPP2R5A, PPP2R5C, RAF1, RHOA]

GO:0004114	Oocyte meiosis	KEGG_01.03.2017	63.0*E* − 6	6.00	[IGF1R, PPP1CB, PPP2CB, PPP2R5A, PPP2R5C, PPP3CB]

GO:0004152	AMPK signaling pathway	KEGG_01.03.2017	570.0*E* − 6	5.00	[IGF1R, PPP2CB, PPP2R5A, PPP2R5C, RHEB]

GO:0004261	Adrenergic signaling in cardiomyocytes	KEGG_01.03.2017	140.0*E* − 6	6.00	[PPP1CB, PPP2CB, PPP2R5A, PPP2R5C, TPM1, TPM2]

GO:0004730	Long-term depression	KEGG_01.03.2017	4.6*E* − 3	3.00	[IGF1R, PPP2CB, RAF1]

GO:0005210	Colorectal cancer	KEGG_01.03.2017	4.6*E* − 3	3.00	[CYCS, RAF1, RHOA]
